# The impact of SARS on hospital performance

**DOI:** 10.1186/1472-6963-8-228

**Published:** 2008-11-06

**Authors:** Dachen Chu, Ran-Chou Chen, Chia-Yu Ku, Pesus Chou

**Affiliations:** 1 Community Medicine Research Center and Institute of Public Health, National Yang-Ming University, Taipei, Taiwan; 2 Department of Biomedical Imaging and Radiological Sciences, National Yang-Ming University, Taipei, Taiwan; 3 Taipei City Hospital, 33 Sec. 2 Chun Hwa Road, Taipei, Taiwan

## Abstract

**Background:**

During the SARS epidemic, healthcare utilization and medical services decreased significantly. However, the long-term impact of SARS on hospital performance needs to be further discussed.

**Methods:**

A municipal hospital in Taipei City was shut down for a month due to SARS and then became the designated SARS and infectious disease hospital for the city. This study collected the outpatient, inpatient and emergency service volumes for every year from April to March over four years. Average monthly service amount ± standard deviation were used to compare patient volume for the whole hospital, as well as the outpatient numbers accessing different departments. The ARIMA model of outpatient volume in the pre-SARS year was developed.

**Results:**

The average monthly service volume of outpatient visits for the base year 2002 was 52317 ± 4204 visits per month, and number for 2003 and the following two years were 55%, 82% and 84% of the base year respectively. The average emergency service volume was 4382 ± 356 visits per month at the base year and this became 45%, 77% and 87% of the base year for the following three years respectively. Average inpatient service volume was 8520 ± 909 inpatient days per month at the base year becoming 43%, 81% and 87% of the base year for the following three years respectively. Only the emergency service volume had recovered to the level of a non-significant difference at the second year after SARS. In addition, the departments of family medicine, metabolism and nephrology reached the 2002 patient number in 2003. The ARIMA (2,1,0) model was the most suitable for outpatient volume in pre-SARS year. The MAPE of the ARIMA (2,1,0) model for the pre-SARS year was 6.9%, and 43.2%, 10.6%, 6.2% for following 3 years.

**Conclusion:**

This study demonstrates that if a hospital is completely shut down due to SARS or a similar disease, the impact is longer than previous reported and different departments may experience different recover periods. The findings of this study identify subspecialties that are particularly vulnerable in an infectious disease designated hospital and such hospitals need to consider which subspecialties should be included in their medical structure.

## Background

Severe Acute Respiratory Syndrome (SARS) is a viral respiratory disease caused by a coronavirus (SARS-CoV). SARS has caused a significant impact on psychosocial and legislative regulation [1-4]. SARS brought about not only relatively discernable economic losses [5], but also observable damage to healthcare organizations, and this has resulted in a lower healthcare utilization rate [6,7].

During the SARS epidemic, there were many reports that looked into healthcare utilization and decreases in medical service volume [8]. However, most of them explored only one department of the hospital or over a very short period of time. No reports have studied the influence on whole hospital performance and followed the long-term impact on the recovery. A municipal hospital in Taipei was shut down for a month due to SARS [3], and afterwards became the designated SARS and infectious disease hospital for the city in addition to its general regional hospital's character. This study collects the service volumes of all departments in this hospital from one year before and for three years after the SARS outbreak. No similar study has been published previously.

## Methods

The municipal hospital in Taipei City studied here is a general and teaching hospital with 450 beds. The departments in this hospital include internal medicine, surgery, OBS/GYN, pediatrics, radiology and pathology. The hospital underwent a SARS outbreak during April 2003. Fifty-nine hospital workers were infected and this led to seven deaths [1,3]. To halt the outbreak of SARS infection, the hospital was shut down to allow evacuation of hospitalized patients and workers gradually over 10 days. Twenty-five days after that, the hospital was fully vacated and sterilization carried out. Outpatient services were first reopened after 35 days of quarantine. Inpatient and emergency services were reopened after 65 days. Subsequently, in addition to its general regional hospital function, the hospital became the infectious disease designated hospital for the Taipei area, and receives infectious disease patients, undergoes preparedness exercises and admits tuberculosis patients.

In order to compare the recovery situation after SARS, this study follows the numbers of outpatients, emergency patients and inpatients from 2002 to 2006. The patient numbers for emergency and outpatient services, and number of patient days for inpatient service were used as quantitative evaluations and are expressed as the average monthly services amount ± standard deviation. The hospital was shut down during April 2003, and therefore the study collected the services volume from every April to next March. Year 2002 was selected as the base year of this study. The service volume of 2003, which is when SARS was occurred, and of the two years after that, were compared to the 2002 data. The study was approved by the ethics committee of the Taipei City Hospital Hoping branch.

Statistically analysis by repeat measurement ANOVA (SPSS for Windows 15.0) was utilized in this study to compare the variation over 4 years. If the service volume for a specific area and year was less than the base year, and showed a significant difference (*p *< 0.05), then this year was considered to be still recovering. Based on this recovery situation, all the departments can be divided into four groups; these are departments that recovered in the year SARS happened, departments that recovered in the first year after SARS, departments that recovered in the second year after SARS, and finally departments still not yet recovered. The autoregressive integrated moving average (ARIMA) model of outpatient volume in the pre-SARS year was developed. Various permutations of the order of correlation and order of integration (I) were computed to choose the optimal combination of parameters based on the mean square error. Partial correlogram graphs and correlograms were used to select the order of moving average (MA) and autoregressive (AR) terms in the model. Based on the best model selected, the mean absolute percentage error (MAPE) was used to measure the quality of fit of the service volume of outpatients during the SARS, and two years after SARS period [9].

During the four years of this study, the economic aspects that affected the hospital's performances remained the same, include beds and medical specialties. Furthermore, the number of similar hospitals in local area did not change. The organization of the healthcare system and its reimbursement still come from the Taiwan National Health Insurance (NHI) system [10]. In addition, there were also no significant policy changes with respect to NHI payment policy in these four years. Moreover, the neighborhood population of this hospital underwent no obvious change (the neighborhood population was 173,637 in 2002 and had become 171,092 in 2006) [11]. However, in addition to the general regional hospital, the hospital was also now designated as an infectious disease hospital.

## Results

**Figure 1:**
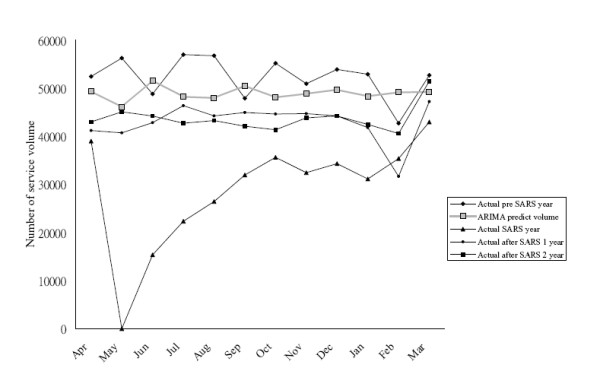
**The ARIMA (2,1,0) model was the most suitable by using the pre-SARS year as the training dataset.** Delayed recovery of out-patient services is noted. The MAPE of the ARIMA (2,1,0) model for the pre-SARS year was 6.9% , 43.2% for the year during SARS attack, 10.6% of the 1^st ^post-SARS year, and improving to 6.2% of the 2^nd ^post-SARS year.

The average monthly number of outpatient visits at base year was 52317 ± 4204. The numbers for the year SARS that happened and the following two years were 55% (28950 ± 11731), 82% (42906 ± 4047) and 84% (43715 ± 2758) of the base year respectively (Table 1). Average emergency numbers was 4382 ± 356 visits per month at base year. This number decreased to 45% (1975 ± 1242) of the base year for the year SARS happened, and came back to 77% (3395 ± 345) during the first year after SARS, and 87% (3824 ± 346) of the base year during the second year after SARS. The average inpatient service volume was 8520 ± 909 inpatient days per month at base year. The number decreased to 43% (3678 ± 2350) of base year for the year SARS happened, and came back to 81% (6863 ± 486) and 87% (7371 ± 471) of the base year for the following two years. Only the emergency service volumes recovered to the level of non-significant difference at the second year after SARS. Services volumes for outpatients and inpatients were still significant different (*p *< 0.05). Using the pre-SARS year as the training dataset in the ARIMA model, the ARIMA IAR (2,1,0) model was the most suitable. Similar result about the delayed recovery of outpatient services is noted (figure 1). The MAPE of the ARIMA (2,1,0) model for the pre-SARS year was 6.94%. The MAPE for the year of SARS was 43.24%, improving to 10.58% for the 1^st ^post-SARS year, and to 6.20% for the 2^nd ^post-SARS year.

**Table 1:** Average monthly service volumes of the outpatient, inpatient and emergency services before, during and after the SARS epidemic

**Year**	**Pre-SARS mean ± SD**	**During SARS mean ± SD**	**%**	**Post SARS (1^st ^yr) mean ± SD**	**%**	**Post SARS (2^nd ^yr) mean ± SD**	**%**
Outpatients^$^	52317 ± 4204	28950 ± 11731*	55	42906 ± 4047*	82	43715 ± 2758*	84
Emergency services^$^	4382 ± 356	1975 ± 1242*	45	3395 ± 345*	77	3824 ± 346	87
Inpatients^$^	8520 ± 909	3678 ± 2350*	43	6863 ± 486*	81	7371 ± 471*	87

* *p *< 0.05, significantly less than the Pre-SARS year (2002)

^$ ^Outpatient and emergency service volumes: number of visits, inpatient service volumes: number of inpatient days.

On examining the various outpatient departments, family medicine, metabolism and nephrology recovered during the year that SARS happened. Family medicine was even higher than the base year during the first and second years after SARS. Nephrology was also higher than the base year during the second year after SARS. The departments that recovered during the first year after SARS included neurology, cardiology, infectious diseases, neurosurgery, urology, plastic surgery, dentistry and psychiatry. Among these, psychiatry was even higher than the base year. Pediatrics recovered during the second year after SARS. The departments that still have not recovered include general surgery, ophthalmology, orthopedics, ENT, internal medicine, pulmonary medicine, gastroenterology, OBS/GYN, dermatology, rehabilitation and Chinese medicine (Table 2). The numbers of physicians were 165 for the base year, 162 for the SARS year, 170 for the first year after SARS, and 173 for the second year after SARS. The numbers of physicians in each department were also similar for these years with differences ranging from 0 to 2 physicians.

**Table 2:** The average monthly numbers for outpatients using different departments. (mean ± SD)

Recovery yr.	Department	Pre-SARS	During SARS	Post SARS (1^st ^yr)	Post SARS (2^nd ^yr)
1^st^-year recovery					
	Nephrology	1522 ± 375	1486 ± 829	1589 ± 703	2855 ± 134 †
	Family medicine	2103 ± 873	1510 ± 797	3674 ± 975 †	3739 ± 945 †
	Metabolism	2151 ± 623	1674 ± 670	2131 ± 266	1894 ± 273
2^nd^-year recovery					
	Psychiatry	1093 ± 298	815 ± 325 *	1341 ± 142 †	1239 ± 131
	Neurosurgery	545 ± 207	355 ± 159 *	626 ± 49	611 ± 59
	Urology	1701 ± 443	1028 ± 446 *	1699 ± 142	1807 ± 132
	Neurology	1900 ± 117	1239 ± 467 *	1877 ± 160	1876 ± 143
	Plastic surgery	329 ± 73	128 ± 87 *	337 ± 51	316 ± 40
	Cardiology	3723 ± 748	2790 ± 1091*	3699 ± 286	3498 ± 270
	Dentist	852 ± 516	462 ± 198 *	638 ± 83	790 ± 161
	Infectious diseases	1144 ± 991	303 ± 286 *	677 ± 94	633 ± 112
3^rd^-year recovery					
	Pediatrics	1867 ± 215	764 ± 387*	1144 ± 190 *	1518 ± 728
No recovery by the 4^th ^year					
	Ophthalmology	4207 ± 443	2627 ± 1032 *	3169 ± 358 *	3578 ± 339 *
	Orthopedics	2808 ± 251	1537 ± 593 *	2274 ± 200 *	2366 ± 142 *
	General surgery	1812 ± 217	982 ± 435 *	1506 ± 192 *	1461 ± 101*
	ENT	3592 ± 410	1750 ± 752 *	2194 ± 213 *	2104 ± 117 *
	Gastroenterology	3071 ± 818	1801 ± 629 *	1911 ± 381 *	1799 ± 243 *
	Dermatology	2244 ± 398	1070 ± 373 *	1720 ± 264 *	1735 ± 132 *
	Chinese medicine	3510 ± 386	1466 ± 805 *	3126 ± 778	2647 ± 281 *
	Rehabilitation	1619 ± 132	702 ± 360*	964 ± 139*	919 ± 83*
	Internal medicine	6414 ± 1159	2380 ± 1073 *	3513 ± 398 *	3542 ± 189 *
	Pulmary medicine	602 ± 175	370 ± 171 *	301 ± 213*	327 ± 75 *
	Obs/Gyn	3177 ± 429	1562 ± 670 *	2392 ± 314 *	2259 ± 691*

* *p *< 0.05: significant less than the pre-SARS year (2002).

† *p *< 0.05: significant higher than the pre-SARS year (2002)

## Discussion

The SARS crisis at this municipal hospital during April 2003 resulted in a reduction in outpatient, emergency and inpatient services to 45%, 55% and 57% respectively, compared to the previous year. The delayed recovery of outpatient service was also noted during and in the first year after SARS by the ARIMA model. After the SARS epidemic, the hospital became the infectious disease designated hospital for the Taipei area in additional to retaining its character as a general regional hospital. Many factors caused the changes in hospital performance, such as patient transfer to other hospitals, reduction of healthcare manpower due to death, sequelae to staff infection and staff turnover. In addition, the publicity related to the change in the hospital status to an infectious disease unit influenced performance. In the few years since SARS, hospital performance has improved continuously. However, only emergency services had recovered by the second year after SARS. Outpatient and inpatient services had still not recovered by 2006.

If we consider outpatient services, in the neighboring countries, certain reports have indicated a 20% to 59% decrease during the SARS epidemic only [12-14]. Vlantis reported that the weekly outpatient clinic attendance showed a decline of 59% and the daily admission rate by 84% for the division of otorhinolaryngology head and neck surgery at an academic tertiary referral hospital in Hong Kong [14]. None of the reported hospitals were shut down during the event. The outpatient service volume in this study shows a longer recovery period and the reason maybe relate to the 35 days of shut down.

There were also huge recovery differences between the different departments even though the physicians' numbers are similar over these four years. Family medicine, metabolism and nephrology departments have recovered quickly. Moreover, patient visits to family medicine and nephrology were even higher than before SARS. This indicates that chronic patients who need long-term treatment tend to go back to their former local hospital to receive their treatments. The patient visits to psychiatry decreased during the year SARS happened, and were obviously increased the year after SARS. This suggests that people may have needed to rely on psychiatry treatment after the SARS epidemic. Outpatient visits to surgery recovered slower. Only urology, plastic surgery and neurosurgery recovered in the year after SARS, and all others have not recovered as yet. Thus, the general public may not wish to receive surgery from an infectious disease designated hospital that once underwent an emergency shut down. As a result, the recovery of inpatient services is definitely affected. Inpatient services usually come from outpatient and emergency transfers. Therefore, recovery of the inpatient service volume is much slower than outpatient and emergency services.

We observe recovery differences across different departments. However, the limitation of this study is that we are unable to explain the failure of certain departments to return to normal service levels in terms of the quantitative and statistical measurements of some variables that may affecting the performance of the hospital. A population based survey of patient's willingness and physicians' attitude such as the KAP (knowledge, attitude, practice) might help in this.

Emergency department (ED) visits during SARS were decreased worldwide when the available literature is considered [6,8,13,15]. The duration of the impact on each hospital as described in these papers is not the same, with one decreased for three months [16], another had recovered by the end of year 2003 after SARS [17], and another had recovered during the second year after SARS [18]. In this study, ED visits only recovered during the second year after SARS. There was no change in the ED numbers in the local area. Some papers have suggested that the decrease in ED visits can have different impacts on different subdivisions. For example, non-critical ED patient visits may decrease more than the critical patient visits [17,19]. One possible reason for the delay in ED service recovery at this hospital is that non-critical patient chose to go to other hospitals, and this needs further research. Alternatively, a patient response to the emergency whole hospital shut down may have occurred or there may be a mixture of both factors.

The impacts caused by SARS were very serious, especially on global economics and health care. In order to control the epidemic, the Department of Health, Executive Yuan, Taiwan, designated some public hospitals to admit major infectious disease patients. This measure had a positive effect on controlling the epidemic [3], and reminds people of the importance that public hospitals have in public health, preventive medicine, and the prevention of infectious diseases. Even three years after SARS, the nation still maintains the ideology of a designated hospital, and has expanded the prevention network national wide. Under the threat of new epidemics, an infectious disease designated hospital has to face the issue of losses in its operating performance. The medical standards and ideology of the designated hospital are always affected.

At present, medical expenditure is increasing dramatically everywhere in the world. It is very important for an infectious disease designated hospital to maintain its self-sufficient operation when the public health budget is under threat. Self-sufficient operation can reduce the need for a government subsidy and allow the maintenance of an adequate number of physicians as well as the quality of medical services at such a general hospital. Although the outpatient and inpatient services did not completely recovered from SARS during first year, the hospital had reached 84% to 86% of the baseline service volume during the second year after SARS. Therefore, the hospital should be capable of self-sufficiency as a designated hospital. In particular, the family medicine, metabolism and nephrology departments were hardly influenced by the shut down. In addition, some other departments had recovered by the second year. Therefore, these departments were able to maintain a sufficient number of physicians in the absence of an infection emergency. If another outbreak of major infectious disease occurs, the physicians in the above mentioned departments would be able to participate directly in prevention tasks. An adequate number of physicians are essential for the success of an infectious disease designated hospital. Thus, the findings of this study should provide a direction for other infectious disease designated hospitals to consider when deciding what subspecialties should be included in their makeup. If an infectious disease designated hospital includes a certain subspecialty and the physicians can operate self-sufficiently during ordinary times, the government only has to subside such a hospital during the year of the epidemic and the year after that. The hospital can become self-sufficient again quickly once everyday operations return.

## Conclusion

In conclusion, this study demonstrates that if the whole hospital is shut down during an infectious disease outbreak, the impact is much longer than other studies have shown. The outpatient and inpatient services in this example had not completely recovered by the second year after SARS. Emergency services were the fastest recovering department, but even this unit did not recover until the second year after SARS. Among outpatient services, family medicine, metabolism and nephrology recovered better than the others. Surgery needs a longer recovery period. This study provides the decision maker with information regards choices when managing an infectious disease designated hospital and is a reference resource for future public policy crisis management decisions during acute infectious disease outbreaks.

## Competing interests

The authors declare that they have no competing interests.

## Authors' contributions

DC conceived of the study, performed the statistical analysis, and wrote the manuscript. RCC participated in study design, coordination, and interpretation of the manuscript. CYK drafted the manuscript and collected data. PC participated in the design of the study, interpretation of the statistical analysis, and coordination. All authors read and approved the final manuscript.

## Pre-publication history

The pre-publication history for this paper can be accessed here:


